# The impact of indigenous practices to promote women's empowerment in agriculture in South Africa

**DOI:** 10.3389/fpubh.2024.1393582

**Published:** 2024-04-30

**Authors:** Lindie von Maltitz, Yonas T. Bahta

**Affiliations:** Department of Agricultural Economics, Faculty of Agricultural and Natural Sciences, University of the Free State, Bloemfontein, South Africa

**Keywords:** engagement, stakeholders, community, political (dis)engagement, culture, indigenous knowledge

## Introduction

Women's empowerment is broadly categorized into three categories: agency—the ability to make decisions; resources—assets, health, and education—the channels through which agency flows; and achievements—economic opportunities and improved social capital—which results from agency (1). Empowerment is also defined as the ability of vulnerable people to have sufficient influence and control over different factors that influence their lives by developing their capabilities and expanding their resources (2).

Different research studies conducted on women's empowerment link the knowledge resource with formal education (3). However, in rural areas where formal education is often limited, indigenous knowledge is essential to the knowledge base. The indigenous knowledge women use in agriculture has yet to be specified in empowerment research studies. Women are typically the custodians of indigenous knowledge and practice in society (4). According to Senanayake [(5), p. 87], indigenous practices “refer to traditional knowledge, skills, and techniques passed down through generations in a particular community or culture.”

Furthermore, Mugabe (6) and Masango [(7), p. 74] defined indigenous knowledge as “knowledge that is held and used by people who identify themselves as indigenous of a place, based on a combination of cultural distinctiveness and prior territorial occupancy relative to a recently arrived population with its own distinct and subsequently dominant culture. This knowledge is gained through past experiences and observations.” Indigenous knowledge and practices differ between areas and communities, and it is essential to comprehend the cultural complexity of its heritage and narrative (8, 9).

Women play an essential role in the global agricultural sector, especially in Sub-Saharan Africa. In rural areas, many households are female-headed and are involved in agriculture to survive and feed their families (10). The FAO (11) showed that in Sub-Saharan, North-East and Northern Africa, more than 45% of the population involved in agriculture are female, and the percentage increases with time. According to StatsSA (12), in South Africa, 41.9% of households are female-headed, with the majority living in rural areas. Women's impact on nutrition, food security, and the economic survival of their families is substantial and often underestimated (13).

In many countries, gender gaps influencing women's empowerment are still prevalent. These include time constraints, income discrimination, confined access to resources, low educational levels, limited decision-making power, excessive workload, and unjust social norms and beliefs (14, 15). Empowering women in agriculture and closing the gender gap in rural societies result in positive change (16). Fletschner and Kenney (17) indicated that women's empowerment has a solid long-term impact on children's health, nutrition, and education, benefiting families and societies.

This opinion aims to highlight the relevance of embracing indigenous practices and knowledge as a knowledge resource for women's empowerment in agriculture.

## Theoretical framework and literature review

Many rural African communities still use indigenous knowledge, particularly for agricultural production. In their review of studies that focused on the use of indigenous knowledge systems by women in rural development, Gwandure and Lukhele-Olorunju (18) and Akullo et al. (8) report on how women have empowered themselves through applying indigenous knowledge in agricultural production. Examples include finding underground water sources through the identification of specific plant species with which women supply their households and producing indigenous crops such as *nyemba* (cowpeas), *nyimo* (Bambara nuts), *mhunga* (millet), *manhanga* (pumpkins), *mashamba* (cattle melon), *mapfunde* (sorghum) to feed their families and to earn an income by selling the extra production. Using a mixture of ash and manure as fertilizer and controlling pests, using local fauna and flora for pest and disease control, reviving depleted soils with specific plants, and formulating deworming for animals from the combination of different plant materials. Indigenous knowledge is also used to predict the weather by observing specific plants and animals (19).

The connection between women's empowerment and the transmission of indigenous knowledge includes other subject areas such as traditional medicine, land use and management, family health care, child-rearing, and survival strategies that collectively enhance sustainable development. In Mali, Henning (20) reported rural women's use of indigenous knowledge of Jatropha to produce curcas oil as raw material, used as a laxative, to produce latex to stop bleeding and against infections, applied as a repellant against mosquitos carrying malaria, soap production and fuel.

The use and transmission of indigenous knowledge, however, has been reported to be declining due to younger generations preferring modern training methods. Limited efficiency has been reported in some cases, and certain legislation prohibits the production of specific plant species (such as cannabis). Many believe it to be too time-consuming (15, 21, 22). Indigenous knowledge is frequently not formally documented and is lost as knowledgeable elders pass away. The increased integration of rural people with the market economy has also been mentioned as a significant role player in the loss of indigenous knowledge, as people can easily find and obtain alternatives (23).

## Research methodology

This opinion paper is based on the existing literature review, the author's previous work on women's empowerment, and indigenous knowledge of the case studies of the South African provinces of the Northern Cape and Eastern Cape, respectively.

Several methods for measuring empowerment have been used over the years, including the Gender Development Index, which considers gender differences in human development, and the Gender Inequality Index, which focuses on gender gaps in health, empowerment, and labor force (21).

Lombardini, Bowman, and Garwood (24) developed an index for Oxfam to measure empowerment in terms of personal, relational, and environmental levels. A Women's Empowerment Index is then customized using context-specific factors in a questionnaire to collect data with specific indicators and weights assigned. Glennerster, Walsh, and Diaz-Martin (25) developed an empowerment measurement that was used to measure young women's empowerment. Biswas and Kabir (26) developed an index to assess women's empowerment based on eleven indicators: mobility, decision-making power, autonomy, ownership of household assets, freedom from domination, awareness, participation in public protests and political campaigns, contribution to family income, reproductive rights, information exposure, and participation in development programs. Formal schooling, media exposure, and indigenous knowledge all provide opportunities for information exposure.

The Women's Empowerment in Agriculture Index was created in 2012 for use in the United States Government's Feed the Future Initiative to assess potential changes in empowerment levels for women in agriculture (14). After implementing the index in projects, researchers discovered it to be overly thorough, with some of the questions difficult to answer and the time span too long for practical implementation in some circumstances. The Abbreviated Women's Empowerment in Agriculture Index (A-WEAI) was then created as a simplified version that could be implemented in the field.

The A-WEAI comprises two sub-indices: the five domains of empowerment index (5DE) and the gender parity index (GPI). The 5DE identifies five areas of empowerment: input into agricultural production decisions, asset ownership and credit access, income control, social capacity, and time allocation. The GPI measures the level of empowerment of a woman compared to that of her husband or another male in the household. Input into production decisions refers not just to household decision-making power but also to the availability of sufficient knowledge to make the proper decisions.

## Results and discussion

A study conducted in the Northern Cape province of South Africa to determine the empowerment level of 93 female smallholder livestock farmers revealed that the average formal education level completed amongst the women was grade 9. They, however, had an average of 12 years of farming experience. When applying the A-WEAI, the results showed that the main components contributing to their disempowerment were their workload (53%), followed by access to and decisions on credit (18%), and lack of ownership of assets (11%). Most respondents answered yes when asked if they had sufficient input into decisions. In this specific study, there was no correlation between the age of the respondents and their empowerment level. Still, there was indeed a positive correlation between the years spent farming and empowerment level, attributed to the fact that experience adds to knowledge, including indigenous knowledge (15).

When interviewed, most of the concerns raised by the women related to funding. However, they also mentioned that a lack of knowledge has dire consequences because of animals dying from disease. They indicated their need for training to increase their knowledge. In South Africa, agricultural advisory professionals employed by the government are responsible for training and providing information to smallholder farmers. Conversely, with a ratio of 1 advisor per 1,053 farmers, the advisory staff needs help to reach farmers on a timely basis (27).

Supporting the use of indigenous knowledge will strengthen and accelerate the government's and the international development community's efforts to increase agricultural productivity. Respective role-players can collaborate to address the decline in the use and transmission of indigenous knowledge due to the preference of younger generations for modern training methods, limited efficiency in some cases, and government restrictions (15, 21, 22).

## Conclusion and recommendations

Indigenous knowledge and practices promote women's empowerment in agriculture but remain marginalized. Better documentation, political recognition, comprehension, and integration of indigenous knowledge with modern science can help promote gender equality and women's empowerment in agriculture. This will contribute to household food security and nutrition to achieve SDGs 2 and 5, which aim to end hunger and poverty, achieve gender equality, and empower all women by 2030.

Knowledge, both formal and indigenous, forms an integral part of women's empowerment. In research studies on women's empowerment in agriculture, the inclusion of indigenous knowledge is yet to be formally specified. This article argues that indigenous knowledge forms an integral part of the knowledge base and should be included in the description of knowledge in the indices used. The affordability and easy access to resources used in indigenous practices in agriculture remain beneficial, especially in poverty-stricken rural areas. The use thereof should be encouraged as it directly influences women's empowerment because it contributes to input in decision-making and allows available funds to be used for other needs.

The formal recording of indigenous knowledge should be encouraged in communities to allow it to be passed on through generations. This could be facilitated by government agricultural advisory professionals who can safely guard and distribute the information. It is a fact that resistance to the use of indigenous knowledge often exists among advisory professionals who prefer to encourage the use of modern-day agricultural practices. However, there is a place for indigenous knowledge as part of the knowledge base, and a combination of these, together with modern-day practices, can be beneficial to empower women.

Given certain complexities, embracing indigenous practices for women's empowerment is a challenging endeavor. The challenge encompasses, but is not limited to, the engagement of stakeholders, including religious, sociocultural, and political actors. To reduce such complexity, we should start engaging and interacting with local and traditional leaders, researchers, government officials, and religious leaders and comprehending the community protocol involved. Respect, ethical engagement, reflexivity, reciprocity, and mutuality are essential.

Most women empowerment indices incorporate formal education. Further research is needed to integrate indigenous knowledge into women's agriculture empowerment indices.

## Author contributions

YB: Conceptualization, Resources, Supervision, Writing – review & editing. LvM: Data curation, Investigation, Writing – original draft.
